# Combination Therapy of Wuweizi (*Schisandrae Chinensis Fructus*) and Dexamethasone Alleviated Dexamethasone-Induced Glucocorticoid Osteoporosis in Rats with Idiopathic Pulmonary Fibrosis

**DOI:** 10.1155/2020/6301697

**Published:** 2020-03-19

**Authors:** Zinan Qin, Siyu Li, Xiaojuan Zhang, Guoxiu Liu, Min Gu, Nan Zhang, Jiao Liu, Zhihong Ji, Keao Li, Yongpeng Han, Huaqiang Zhai

**Affiliations:** ^1^School of Chinese Pharmacy, Beijing University of Chinese Medicine, Beijing, China; ^2^Chongqing Maternal and Child Health Hospital, Chongqing, China; ^3^Xinjiang Medical University, Urumqi, China; ^4^Beijing Hospital of Traditional Chinese and Western Medicine, Beijing, China

## Abstract

**Objective:**

To investigate the therapeutic effect of combined application of Wuweizi *(Schisandrae Chinensis Fructus)* and dexamethasone in rats with idiopathic pulmonary fibrosis (IPF) and the possible protective effect of Wuweizi against dexamethasone-induced glucocorticoid osteoporosis (GIOP).

**Methods:**

There were five groups in this study, including the sham operation group, model group, Wuweizi group, dexamethasone group, and the combination group. A rat IPF model was made by the endotracheal injection of bleomycin. After modeling, rats were given drug interventions for 7 and 28 days. Rats were sacrificed for pathological morphology examination of the bone and lung and quantitative determination of biochemical markers of bone metabolism and angiogenesis-related cytokine to observe therapeutic efficacy on the 7^th^ and 28^th^ day. ELISA was used for the quantitative determination of tartrate-resistant acid phosphatase (TRACP), bone alkaline phosphatase (BALP), hypoxia-inducible factor (HIF-1*α*), platelet-derived growth factor (PDGF), pigment epithelium-derived factor (PEDF), and endostatin in serum. The concentrations of calcium (Ca) and phosphorus (P) were detected with the automatic biochemical analyzer.

**Results:**

After drug interventions for 7 and 28 days, alveolitis and pulmonary fibrosis in treatment groups showed significant improvement compared with those in the model group (*P* < 0.05). Bone histopathological figures showed severely damaged trabecular bone and bone marrow cavity in the dexamethasone group, but it was significantly alleviated in the combination group. The concentrations of BALP and Ca in the combination group were significantly higher than those in the dexamethasone group after treatment, while the concentrations of TRACP and P were lower than those in the dexamethasone group (*P* < 0.05). Bone histopathological figures showed severely damaged trabecular bone and bone marrow cavity in the dexamethasone group, but it was significantly alleviated in the combination group. The concentrations of BALP and Ca in the combination group were significantly higher than those in the dexamethasone group after treatment, while the concentrations of TRACP and P were lower than those in the dexamethasone group (*α*), platelet-derived growth factor (PDGF), pigment epithelium-derived factor (PEDF), and endostatin in serum. The concentrations of calcium (Ca) and phosphorus (P) were detected with the automatic biochemical analyzer. *P* < 0.05). Bone histopathological figures showed severely damaged trabecular bone and bone marrow cavity in the dexamethasone group, but it was significantly alleviated in the combination group. The concentrations of BALP and Ca in the combination group were significantly higher than those in the dexamethasone group after treatment, while the concentrations of TRACP and P were lower than those in the dexamethasone group (*P* < 0.05). Bone histopathological figures showed severely damaged trabecular bone and bone marrow cavity in the dexamethasone group, but it was significantly alleviated in the combination group. The concentrations of BALP and Ca in the combination group were significantly higher than those in the dexamethasone group after treatment, while the concentrations of TRACP and P were lower than those in the dexamethasone group (*α*), platelet-derived growth factor (PDGF), pigment epithelium-derived factor (PEDF), and endostatin in serum. The concentrations of calcium (Ca) and phosphorus (P) were detected with the automatic biochemical analyzer.

**Conclusions:**

The combination therapy of Wuweizi and dexamethasone effectively treated IPF rats by regulating angiogenesis, meanwhile distinctly alleviating dexamethasone-induced GIOP.

## 1. Introduction

Idiopathic pulmonary fibrosis (IPF) is a specific form of chronic, progressive, fibrosing interstitial pneumonia of unknown cause that has few treatment options [1, 2], and its pathological change is characterized by inflammatory response and progressive fibrosis in the injured lung [3]. Therefore, it seriously affects the quality of patients' life. Dexamethasone is a kind of glucocorticoid that was widely used for the treatment of IPF [4–6], but its clinical application is limited due to its adverse drug reactions. GIOP is one of the common and frequent side effects of dexamethasone [7]. The 2010 ACR GIOP consensus clearly stated that osteoporosis should be prevented regardless of dose size for patients receiving glucocorticoids [8]. Scholars have strived to find strategies to prevent adverse drug reactions of dexamethasone. Chinese herbal medicine has been widely used to treat GIOP in China recently. Of note, Wuweizi (*Schisandrae Chinensis Fructus*) has been proven to be effective in the treatment of IPF and GIOP. When multiple drugs are used in combination, especially western medicine and Chinese herbal medicine, they often compete for the pharmacological targets with each other and trigger drug-drug interactions (DDIs) and, finally, attenuate or increase their efficacy and toxicity [9]. Therefore, we used Wuweizi and dexamethasone together in IPF rats to explore the therapeutic effect of combination therapy, meanwhile investigating whether combination therapy can alleviate dexamethasone-induced adverse drug reactions.

Wuweizi (*Schisandrae Chinensis Fructus*), the dried ripe fruit of *Schisandra Chinensis* (Turcz.) Baill., has been indexed in the Pharmacopoeia of China. It is rich in sugar, flavonoids, and lignans, of which lignans are considered major active ingredients of it, including schisandrin A, schisandrin B, schisandrin C, schisandrol A, and schisandrol B [10]. Wuweizi was widely used for liver protection and as anti-inflammatory and antitumor in clinical cases [10–12]. Our recent studies have found that Wuweizi treated IPF rats effectively by regulating the chemotactic migration of cells, angiogenesis, and AM/TGF*β*-1/Smad signaling pathways [13–15]. Furthermore, many studies documented that Wuweizi has a protective effect on bone [16–19] and can be a promising herb in the treatment of osteoporosis [19]. Plentiful clinical and experimental studies in China also have found that Wuweizi can effectively alleviate GIOP [20–22].

The pathological changes of GIOP include damaged bone tissue and abnormal bone metabolism [23], so we studied the pathological morphology of bone tissue and levels of biochemical markers of bone metabolism in IPF rats after intervention with dexamethasone to investigate the protective effect of Wuweizi against dexamethasone-induced GIOP in this work. Abnormal angiogenesis is a possible pathogenesis of IPF [24, 25]. According to the previous researches in our laboratory, high levels of proangiogenesis factors and low levels of antiangiogenesis factors were observed in IPF rats [14, 26, 27]. Therefore, we detected the concentrations of hypoxia-inducible factor (HIF-1*α*), platelet-derived growth factor (PDGF), pigment epithelium-derived factor (PEDF), and endostatin to investigate the efficacy of combination therapy in our study. Overall, to investigate the potential effect of combination therapy to treat IPF rats and alleviate dexamethasone-induced adverse drug reaction, we studied the pathological morphology of lung tissue and bone tissue and the concentrations of angiogenesis factors and biochemical markers of bone metabolism in IPF rats after distinct drug interventions in our work. To a certain degree, this research could provide some active reference in the combination of Chinese herbs and western medicine in clinical practices to promote relevant research and practice.

## 2. Methods and Materials

### 2.1. Drugs and Reagents

Wuweizi (*Schisandrae Chinensis Fructus*) was purchased from Beijing Taiyang Shukang Traditional Chinese Medicine Pieces Factory (Beijing, China) and was identified by Prof. Huaqiang Zhai. Dexamethasone acetate tablets were obtained from Tianjin Lisheng Pharmaceutical Co., Ltd. (Tianjin, China). Bleomycin hydrochloride for injection was obtained from Nippon Chemical Co., Ltd. Wuweizi decoction was prepared by boiling Wuweizi in 8 and 4 times water for 30 and 20 min, respectively, concentrated at 1 g crude drug/ml, and stored at 4°C.

### 2.2. Animals

74 specific pathogen-free (SPF) adult male Wistar rats (6 weeks of age, weighing 185-215 g) were purchased from Beijing Weitong Lihua Experimental Animal Technology Co., Ltd., (Permission No. SCXK (Beijing) 2012-0001). Rats were housed at a controlled temperature and had free access to food and tap water. This study conforms to the ethical review of Beijing University of Chinese Medicine. All animals used in the study received humane care according to institutional animal care guidelines.

### 2.3. Modeling and Drug Administration

The animals were randomly divided into five groups (5 rats in the sham operation group and 8 in each of the other groups). A rat IPF model was made by endotracheal injection of bleomycin referring to Szapiel's method [28]: the rats were anesthetized by intraperitoneally injecting with 1% sodium pentobarbital (30 mg/kg). After disinfecting the skin with ethanol, the neck was cut open, and the trachea exposed, then bleomycin hydrochloride (7 mg/kg) was injected at 2 ml/h through a microinjection pump into the lungs. Rotating rat board to ensure a uniform drug distribution in the lungs and injecting 80000 U/day sodium penicillin to prevent infection after suturing the muscle and skin were indispensable. Rats in the sham operation group underwent the same surgery except bleomycin hydrochloride which was replaced with 0.9% physiological saline.

After modeling, rats in different groups were given different drug interventions by intragastric administration: rats in the sham operation group and model group were given 9% normal saline (5 ml/kg); the Wuweizi decoction (5 ml/kg) was given to the Wuweizi group; the dexamethasone group was treated with dexamethasone suspension (0.5 mg/kg); and the combination group of Wuweizi and dexamethasone was firstly given Wuweizi decoction (5 ml/kg), then dexamethasone suspension (0.5 mg/kg). The frequency of administration was one time per day, and the duration was 28 days.

### 2.4. Pathological Morphology Examination

Rats were sacrificed on the 7^th^ and 28^th^ day after drug administration for obtaining abdominal aortic blood and lung tissue, respectively. Additionally, rats except in the Wuweizi group were also used for obtaining femur on the 28^th^ day. The lower lobe of the left lung was removed and fixed in 10% neutral formalin solution. Meanwhile, the left femur was peeled and fixed in neutral formaldehyde solution and decalcified with neutral EDTA, and the cancellous bone area of the articular cartilage was sliced for examination. Pathological changes in lung tissue and bone tissue were observed with H&E staining under an ordinary light microscope (Nikon microscope, ECLIPSELV100POL/50IPOL Japan).

The classification of alveolitis and pulmonary fibrosis referring to Szapiel's method [28] is as follows: (1) alveolitis classification: no alveolitis (-), mild alveolitis (+; affected area < 20%), moderate alveolitis (++; affected area 20-50%), and severe alveolitis (+++; affected area > 50%) and (2) pulmonary fibrosis classification: no pulmonary fibrosis (-), light degree of pulmonary fibrosis (+; lesion range < 20% in the whole lung), moderate pulmonary fibrosis (++; lesion range 20-50% in the whole lung), and severe pulmonary fibrosis (+++; lesion range 50% in the whole lung, accompanied by alveolar fusion and lung parenchyma structural disorder).

### 2.5. Quantitative Determination of HIF-1*α*, PDGF, PEDF, Endostatin, BALP, and TRACP

The concentrations of HIF-1*α*, PDGF, PEDF, endostatin, BALP, and TRACP in serum were detected by ELISA. ELISA kits were purchased from Shanghai Lanji Biotechnology Co., Ltd. (Shanghai, China, batch No. 20150918), and the whole experimental procedures were performed in strict accordance with the kit instructions.

### 2.6. Quantitative Determination of Ca and P

The concentrations of Ca and P in serum were detected with an automatic biochemical analyzer according to the protocols. Kits were purchased from Nanjing Jiancheng Bioengineering Institute (Nanjing, China, batch No. 20150915 and No. 20150918).

### 2.7. Statistical Analysis

Data were expressed as mean ± standard deviation ($\bar{x}$±𝑠) and analyzed with SPSS 17.0 statistical software (Bizinsight Information Technology Co., Ltd., Beijing, China). Independent sample *t*-test (parameter test) was conducted when the two groups were both subject to a normal distribution (*P* > 0.05) and homogeneity of variance (*P* > 0.05); if not satisfying homogeneity of variance (*P* < 0.05), *t*-test is conducted. Values of *P* < 0.05 and *P* < 0.01 were considered to indicate statistically significant differences and extremely significant differences between groups, respectively.

## 3. Results

### 3.1. Lung Tissue in IPF Rats by H&E Staining

**Figure 1:**
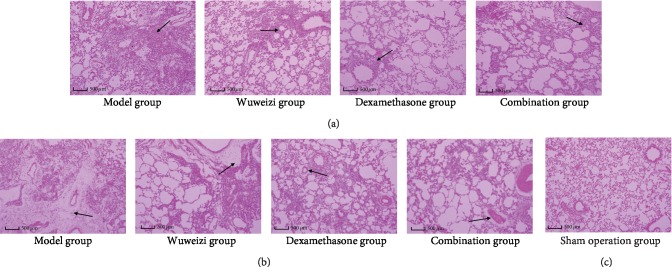
Histopathological images of lung tissues in rats during drug interventions: (a) 7 days after drug treatment, (b) 28 days after drug treatment, and (c) the sham operation group. Arrows, respectively, point to inflammatory cell infiltration and aggregation of fibroblasts at different time points. The scale bars were 500 *μ*m.

The sham operation group demonstrated intact and clear alveolar structure, and there were no fusion, no thickening of the alveolar septum, no congestion, no edema, no inflammation, and no fibrosis (Figure 1(c)). On the 7^th^ day, inflammatory cell infiltration was observed in the visual field in the model group, and we also observed that the neutrophils increased, the alveolar wall was thickened, and the alveolar cavity was broken and merged to form a sizeable pulmonary vesicle; meanwhile, there was no significant fibrosis (Figure 1(a)). Treatment groups presented the defective alveolar structure and inflammatory cell infiltration with varying degrees, respectively; however, it was lighter than those in the model group (Figure 1(a)). The inflammatory cell infiltration in the model group on the 28^th^ day was slightly reduced, compared with that on the 7^th^ day, but the alveolar structure still was disordered, the alveolar wall was damaged, and there were large numbers of fibroblasts aggregated in the pulmonary interstitium (Figure 1(b)). Pulmonary interstitial fibrosis with a varying degree was observed in three treatment groups, but it was lighter than that in the model group (Figure 1(b)).

According to scores of alveolitis and pulmonary fibrosis, alveolitis and pulmonary fibrosis in the model group were more severe than those in the sham operation group. After drug interventions for 7 and 28 days, scores of alveolitis and pulmonary fibrosis in three treatment groups both showed significant differences compared with those in the model group (*P* < 0.05) (Table 1). The dexamethasone group and combination group significantly prevented alveolitis and pulmonary fibrosis in the rats.

### 3.2. Bone Tissue in IPF Rats by H&E Staining

**Figure 2:**
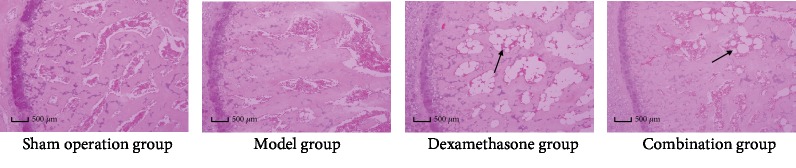
Histopathological images of left femur tissues in rats. Arrows point to the medullary cavity of bones. The scale bars were 50 *μ*m.

Compared with the sham operation group and the model group, the number of bone trabeculae in the dexamethasone group was significantly decreased, the medullary cavity of bones was enlarged, and a large blank area appeared in the dexamethasone group. However, the number of bone trabeculae in the combination group was significantly increased, and the medullary cavity of bones was smaller than that in the dexamethasone group (Figure 2).

### 3.3. Concentrations of HIF-1*α*, PDGF, PEDF, and Endostatin in IPF Rats

**Figure 3:**
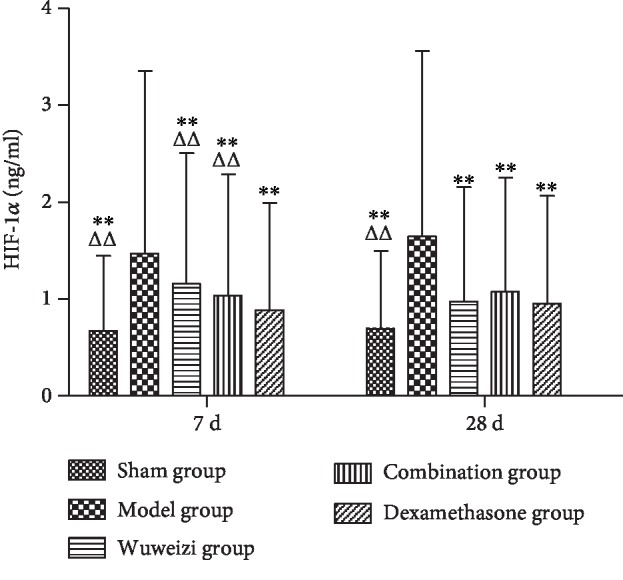
Concentrations of HIF-1*α* in lung tissues of rats before and after drug interventions. ^∗^*P* < 0.05 and ^∗∗^*P* < 0.01, compared with the model group; ^△^*P* < 0.05 and ^△△^*P* < 0.01, compared with the dexamethasone group.

**Figure 4:**
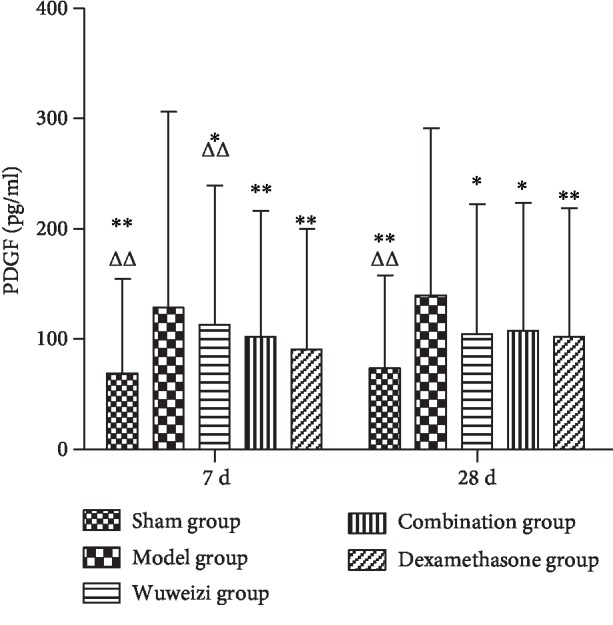
Concentrations of PDGF in lung tissues of rats before and after drug interventions. ^∗^*P* < 0.05 and ^∗∗^*P* < 0.01, compared with the model group; ^△^*P* < 0.05 and ^△△^*P* < 0.01, compared with the dexamethasone group.

**Figure 5:**
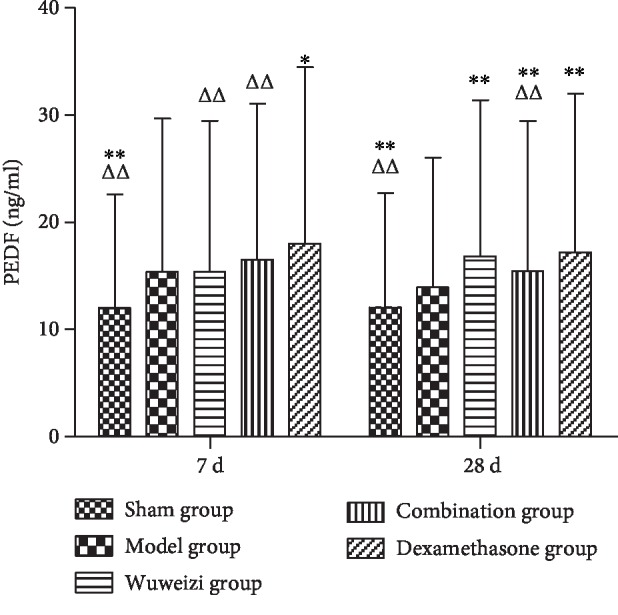
Concentrations of PEDF in lung tissues of rats before and after drug interventions. ^∗^*P* < 0.05 and ^∗∗^*P* < 0.01, compared with the model group; ^△^*P* < 0.05 and ^△△^*P* < 0.01, compared with the dexamethasone group.

**Figure 6:**
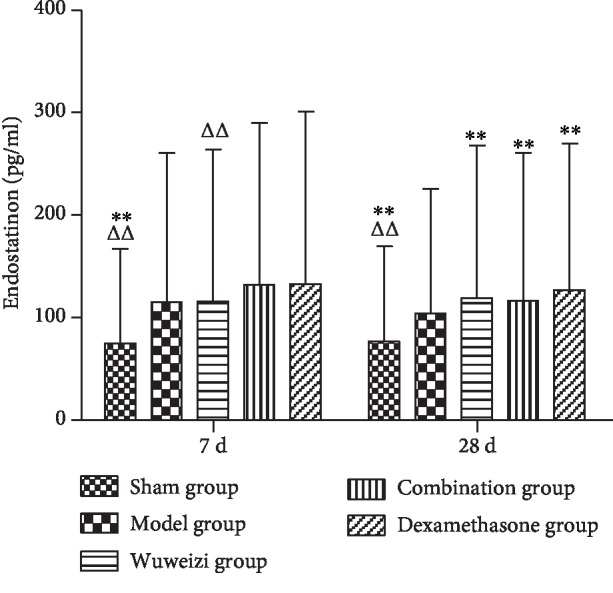
Concentrations of endostatin in lung tissues of rats before and after drug interventions. ^∗^*P* < 0.05 and ^∗∗^*P* < 0.01, compared with the model group; ^△^*P* < 0.05 and ^△△^*P* < 0.01, compared with the dexamethasone group.

The concentrations of HIF-1*α*, PDGF, PEDF, and endostatin in the sham operation group, Wuweizi group, dexamethasone group, and combination group all demonstrated significant differences from the model group. The concentrations of HIF-1*α*, PDGF, PEDF, and endostatin in the sham operation group were all significantly lower than those in the model group on the 7^th^ and 28^th^ day (*P* < 0.01). After drug interventions for 7 and 28 days, the concentrations of HIF-1*α* in three treatment groups were significantly lower than those in the model group (*P* < 0.01). However, the concentration of HIF-1*α* in the combination group was significantly higher than that in the dexamethasone group on the 7^th^ day (*P* < 0.01) (Figure 3). The concentrations of PDGF in three treatment groups were significantly lower than those in the model group (*P* < 0.05) after drug interventions for 7 and 28 days (Figure 4). The concentrations of PEDF in three treatment groups were all higher than those in the model group, especially on the 28^th^ day (*P* < 0.01). However, the concentrations of PEDF in the combination group on the 7^th^ and 28^th^ day were lower than those in the dexamethasone group (*P* < 0.05) (Figure 5). The concentrations of endostatin in three treatment groups were all higher than those in the model group after drug interventions, especially on the 28^th^ day (*P* < 0.01) (Figure 6).

### 3.4. Concentrations of BALP, TRACP, Ca, and P in IPF Rats

**Figure 7:**
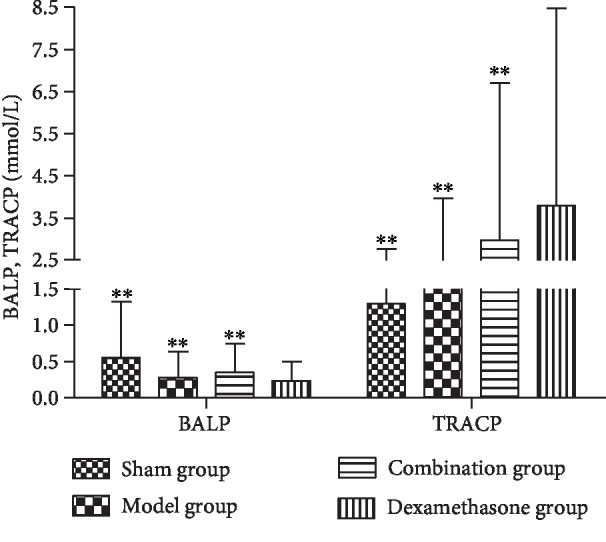
Concentrations of BALP and TRACP in bone tissues of IPF rats before and after drug interventions. ^∗∗^*P* < 0.01, compared with the dexamethasone group.

**Figure 8:**
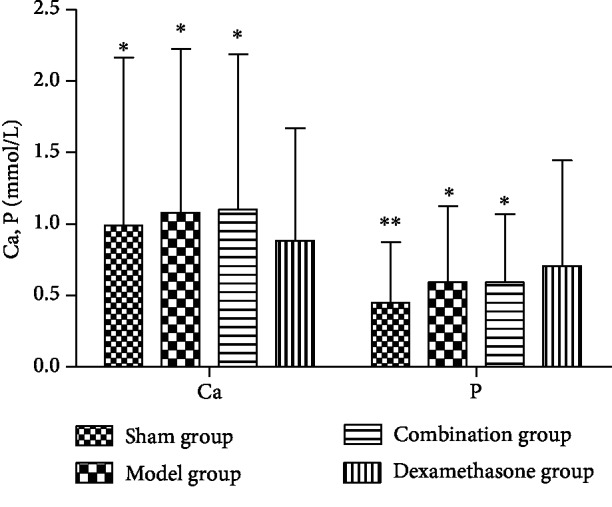
Concentrations of Ca and P in bone tissues of IPF rats before and after drug interventions. ^∗^*P* < 0.05 and ^∗∗^*P* < 0.01, compared with the dexamethasone group.

The concentrations of BALP, TRACP, Ca, and P in the sham operation group, model group, and combination group all demonstrated significant differences from the dexamethasone group. The concentrations of BALP and Ca in the dexamethasone group were significantly lower compared with those in the sham operation group and the model group (*P* < 0.05). Reversely, the concentrations of TRACP and P in the dexamethasone group were higher than those in the sham operation group and the model group (*P* < 0.05). However, the combination therapy significantly increased the concentrations of BALP and Ca and decreased the concentrations of TRACP and P, compared with the dexamethasone group (*P* < 0.05) (Figures 7 and 8).

## 4. Discussions

IPF seriously affects the life of patients. There has been no specific medicine for IPF treatment so far, although IPF treatment guidelines have been continuously updated and improved [5, 6]. Dexamethasone is a classical drug in the treatment of IPF, but chronic use often causes some side effects such as GIOP. Effective management of dexamethasone-induced adverse drug reactions remains to be studied. CYP enzyme-mediated DDIs between multiple drugs often lead to attenuation or enhancement of efficacy and toxicity of drugs. With the widespread use of Chinese herb medicine in recent years, it has shown great value in clinical treatment, so more and more researchers pay attention to integrated Chinese and western medicine in the treatment, in order to increase the efficacy and reduce adverse reactions [29, 30]. We found that both dexamethasone and Wuweizi can induce the activity of the CYP3A4 enzyme [31–33]. Therefore, we hypothesized that Wuweizi might alleviate dexamethasone-induced GIOP by triggering CYP-mediated DDIs in the combination therapy. Our previous studies have found the promising efficacy of Wuweizi in IPF rats [14, 15, 27]. Moreover, it is also a promising herb in the treatment of osteoporosis [19]. Kim et al. proved that Wuweizi attenuated the pathological changes in femur head and condyles of OVX mice via activation of estrogen receptors and enhanced level of serum estrogen [16]. He et al. found that SA, one kind of lignan extracted from Wuwezi, inhibited osteoclast formation by involving in RANKL signaling pathways and decreasing osteolysis area, osteoclasts activity, and osteoclasts number [17]. Caichompoo et al. demonstrated the positive effect of Wuweizi on osteoblasts [18]. Therefore, on the basis of previous studies, we used Wuweizi and dexamethasone in combination in IPF rats to investigate whether the combination therapy could treat IPF rats and alleviate dexamethasone-induced GIOP.

Pulmonary fibrosis is characterized by an inflammatory response and progressive fibrosis in the injured lung tissue [3]. In our study, inflammatory response and fibrosis both appeared at different time points: on the 7^th^ day, the main manifestation was alveolitis, and then on the 28^th^ day, obvious fibrosis appeared in the model group. According to H&E staining, scores of alveolitis and fibrosis in the sham operation group were significantly lower than those in the model group (*P* < 0.05), which indicates that the IPF model was successful. The scores in the model group and treatment groups both presented significant differences (*P* < 0.05) at different time points, indicating that Wuweizi, dexamethasone, and their combined application both can improve the pathological changes of lung tissue and treat IPF rats effectively, which is consistent with previous studies.

The expression of angiogenesis factors is abnormal in IPF. Therefore, we observed the concentrations of the angiogenesis-related factors to evaluate the therapeutic effect of distinct therapeutic methods in IPF rats. HIF-1*α* is a proangiogenesis factor, whose concentration would elevate in hypoxia. Repair and hyperplasia in the injured lung tissue of IPF patients often cause inflammatory cell infiltration, resulting in gas exchange disorder and hypoxia, finally, increasing the level of HIF-1*α* [34]. Ma AP et al. reported that abnormal activation of the PI3K/AKT/HIF-1*α* signaling pathway accelerated the progression of pulmonary fibrosis [35]. As a classical proangiogenesis factor, PDGF is a specific mitogenic factor and chemokine of vascular endothelial cells and fibroblasts, and it can effectively stimulate fibroblast division and proliferation and significantly increase the expression of matrix metalloproteinase (MMP) and tissue inhibitor of metalloproteinase (TIMP), thus inducing angiogenesis. Studies have shown that the level of PDGF was significantly elevated in IPF patients, and it was closely related to the severity of illness [36, 37]. In our study, the concentrations of HIF-1*α* and PDGF in the model group significantly increased compared with those in the sham operation group on the 7^th^ day (*P* < 0.01), indicating that angiogenesis was activated abnormally in the damaged lungs. After distinct treatments, the concentrations of HIF-1*α* and PDGF in each treatment group were significantly lower than those in the model group (*P* < 0.01), indicating Wuweizi, dexamethasone, and the combined therapy can remedy IPF through inhibiting vascular overgrowth. PEDF, the widely known angiogenesis inhibitor, is a protein that inhibits angiogenesis by inducing endothelial cell apoptosis and blocking VEGF-related signaling pathways [38]. Endostatin is an endogenous angiogenesis inhibitor, and it can inhibit angiogenesis by blocking Wnt/*β*-actin, VEGF, and other signaling pathways and reducing cell proliferation and migration [39]. We observed that the concentrations of PEDF and endostatin in the model group significantly decreased compared with those in the sham operation (*P* < 0.01). However, their levels in treatment groups were significantly higher than those in the model group (*P* < 0.05). Therefore, we concluded that the combination therapy treated IPF rats effectively by regulating the balance of proangiogenesis factors and antiangiogenesis factors to normal, indicating that combination therapy of Chinese herbs and western medicine has an optimistic effect in IPF rats.

GIOP is a typical adverse drug reaction of dexamethasone. Dexamethasone usually decreases the volume of trabecular bone, the content of mineral in bone, and the activity of alkaline and acid phosphatase after long-term use, ultimately, inducing GIOP [40]. Therefore, thinning bone trabeculae, reduced volume of trabeculae, increased bone resorption lumen, and unbalanced bone metabolism were observed in GIOP rats; meanwhile, the osteoblasts will decrease, but the osteoclasts will increase [23, 41]. In order to investigate the bone protection effect of the combination therapy in IPF rats, we studied the histopathology of bone and the concentrations of biochemical markers of bone metabolism in IPF rats after intervention with dexamethasone for 28 days. According to H&E staining, the dexamethasone group demonstrated significantly reduced trabecular bone and sparsely fractured and enlarged bone marrow cavity. However, these were significantly alleviated in the combination group. The femoral pathological examination indicated that dexamethasone causes bone toxicity in IPF rats, and Wuweizi exactly can alleviate dexamethasone-induced adverse drug reaction when it is used in combination with dexamethasone.

TRACP is a specific indicator of bone resorption and can be a monitoring indicator for osteoporosis. BALP is an extracellular enzyme of osteoblasts, and its level can reflect the activity of osteoblast and the state of bone metabolism. Furthermore, quantitative determination and dynamic observation of BALP in serum are helpful in the diagnosis and treatment monitoring of osteoporosis [42–44]. Ca is important for the formation of bone and is the basis of prevention and treatment of osteoporosis; meanwhile, the appropriate ratio of Ca to P in bone is vital for bone metabolism [45]. Therefore, detecting the concentrations of Ca and P in the blood has a vital clinical value for monitoring bone metabolism. In this study, we observed decreased concentrations of BALP and Ca and increased concentrations of TRACP and P in the dexamethasone group, indicating that dexamethasone caused abnormal bone metabolism in IPF rats. Compared with the dexamethasone group, the concentrations of BALP and Ca significantly increased but the concentrations of TRACP and P decreased in the combination therapy group (*P* < 0.05), which is in accord with our hypothesis that the combination therapy of Wuweizi and dexamethasone in IPF rats can alleviate dexamethasone-induced GIOP.

## 5. Conclusion

In our study, we found that the combination therapy of Wuweizi and dexamethasone in IPF rats can not only treat pulmonary fibrosis effectively by regulating angiogenesis but also alleviate dexamethasone-induced GIOP. The results of this study revealed that the rational combination use of multiple drugs guided by scientific researches and clinical data could effectively improve the efficacy and reduce the adverse drug reactions of drugs. Therefore, it is meaningful and essential to conduct researches concerning interactions of drugs, especially between Chinese herbs and western medicine. Although our experimental research achieved positive results, the results obtained in animal models must be confirmed in controlled clinical trials in patients. More scientific, rational, and standard researches and practices about combination applications of multiple drugs require a worldwide effort.

## Figures and Tables

**Table 1:** Classification results of alveolar inflammation and fibrosis at different time points.

Group	7-day alveolar inflammation					28-day fibrosis classification				
	N	(-)	(+)	(++)	(+++)	*N*	(-)	(+)	(++)	(+++)
Sham operation group	5	4	1	0	0	5	4	1	0	0
Model group	8^∗^	0	1	1	6	8^∗^	0	0	2	6
Wuweizi (*Schisandrae Chinensis Fructus*) group	8^∗^^△^	0	1	3	4	8^∗^^△^	0	3	3	2
Dexamethasone group	8^∗^^△^	0	3	4	1	8^∗^^△^	0	2	4	2
Combination group	8^∗^^△^	0	4	3	1	8^∗^^△^	0	2	3	3

^∗^ *P* < 0.05, compared with the sham operation group; ^△^*P* < 0.05, compared with model group.

## Data Availability

The data and materials supporting the conclusions of this article are included in the article.
